# Efficacy and Safety of Tixagevimab/Cilgavimab to Prevent COVID-19 (Pre-Exposure Prophylaxis): A Systematic Review and Meta-Analysis

**DOI:** 10.3390/diseases10040118

**Published:** 2022-12-01

**Authors:** Saad Alhumaid, Abbas Al Mutair, Jalal Alali, Nourah Al Dossary, Sami Hussain Albattat, Sarah Mahmoud Al HajjiMohammed, Fatimah Saad Almuaiweed, Maryam Radhi AlZaid, Mohammed Jaber Alomran, Zainab Sabri Alqurini, Ahmed Abduljalil Alsultan, Thamer Saeed Alhajji, Sukainah Mohammad Alshaikhnasir, Ali Al motared, Koblan M. Al mutared, Khalid Hajissa, Ali A. Rabaan

**Affiliations:** 1 Administration of Pharmaceutical Care, Al-Ahsa Health Cluster, Ministry of Health, Al-Ahsa 31982, Saudi Arabia; 2 Research Center, Almoosa Specialist Hospital, Al-Ahsa 36342, Saudi Arabia; 3 College of Nursing, Princess Norah Bint Abdulrahman University, Riyadh 11564, Saudi Arabia; 4 School of Nursing, Wollongong University, Wollongong, NSW 2522, Australia; 5 Department of Nursing, Prince Sultan Military College, Dharan 34313, Saudi Arabia; 6 Internal Medicine Department, King Fahad Hofuf Hospital, Ministry of Health, Al-Ahsa 36441, Saudi Arabia; 7 General Surgery Department, Alomran General Hospital, Ministry of Health, Al-Ahsa 36358, Saudi Arabia; 8 Division of Haematology and Oncology, Pediatric Department, Maternity and Children Hospital, Ministry of Health, Al-Ahsa 36422, Saudi Arabia; 9 Pharmacy Department, Prince Saud Bin Jalawi Hospital, Ministry of Health, Al-Ahsa 36424, Saudi Arabia; 10 Pharmacy Department, Aljafr General Hospital, Ministry of Health, Al-Ahsa 7110, Saudi Arabia; 11 Medical Department, Aljafr Hospital, Al-Ahsa 7110, Saudi Arabia; 12 Pharmacy Department, Prince Sultan Cardiac Center, Ministry of Health, Al-Ahsa 36441, Saudi Arabia; 13 Pharmacy Department, Maternity and Children Hospital, Ministry of Health, Dammam 32253, Saudi Arabia; 14 Pharmacy Department, Eradah Complex and Mental Health, Ministry of Health, Najran 66248, Saudi Arabia; 15 Administration of Pharmaceutical Care, Ministry of Health, Najran 66255, Saudi Arabia; 16 Department of Medical Microbiology & Parasitology, School of Medical Sciences, Universiti Sains Malaysia, Kubang Kerian 16150, Malaysia; 17 Molecular Diagnostic Laboratory, Johns Hopkins Aramco Healthcare, Dhahran 31311, Saudi Arabia; 18 College of Medicine, Alfaisal University, Riyadh 11533, Saudi Arabia; 19 Department of Public Health/Nutrition, The University of Haripur, Haripur 22620, Pakistan

**Keywords:** COVID-19, efficacy, evusheld, safety, SARS-CoV-2, tixagevimab/cilgavimab, systematic review, meta-analysis

## Abstract

Background: Tixagevimab/cilgavimab (TGM/CGM) are neutralizing monoclonal antibodies (mAbs) directed against different epitopes of the receptor-binding domain of the SARS-CoV-2 spike protein that have been considered as pre-exposure prophylaxis (PrEP). Objectives: This study seeks to assess the efficacy and safety of TGM/CGM to prevent COVID-19 in patients at high risk for breakthrough and severe SARS-CoV-2 infection who never benefited maximally from SARS-CoV-2 vaccination and for those who have a contraindication to SARS-CoV-2 vaccines. Design: This study is a systematic review and meta-analysis. The Preferred Reporting Items for Systematic reviews and Meta-Analyses (PRISMA) statement was followed. Methods: Electronic databases (PubMed, CINAHL, Embase, medRxiv, ProQuest, Wiley online library, Medline, and Nature) were searched from 1 December 2021 to 30 November 2022 in the English language using the following keywords alone or in combination: 2019-nCoV, 2019 novel coronavirus, COVID-19, coronavirus disease 2019, SARS-CoV-2, severe acute respiratory syndrome coronavirus 2, tixagevimab, cilgavimab, combination, monoclonal, passive, immunization, antibody, efficacy, clinical trial, cohort, pre-exposure, prophylaxis, and prevention. We included studies in moderate to severe immunocompromised adults (aged ≥18 years) and children (aged ≥12 years) who cannot be vaccinated against COVID-19 or may have an inadequate response to SARS-CoV-2 vaccination. The effect sizes of the outcome of measures were pooled with 95% confidence intervals (CIs) and risk ratios (RRs). Results: Of the 76 papers that were identified, 30 articles were included in the qualitative analysis and 13 articles were included in the quantitative analysis (23 cohorts, 5 case series, 1 care report, and 1 randomized clinical trial). Studies involving 27,932 patients with high risk for breakthrough and severe COVID-19 that reported use of TGM/CGM combination were analyzed (all were adults (100%), 62.8% were men, and patients were mainly immunocompromised (66.6%)). The patients’ ages ranged from 19.7 years to 79.8 years across studies. TGM/CGM use was associated with lower COVID-19-related hospitalization rate (0.54% vs. 1.2%, *p* = 0.27), lower ICU admission rate (0.6% vs. 5.2%, *p* = 0.68), lower mortality rate (0.2% vs. 1.2%, *p* = 0.67), higher neutralization of COVID-19 Omicron variant rate (12.9% vs. 6%, *p* = 0.60), lower proportion of patients who needed oxygen therapy (8% vs. 41.2%, *p* = 0.27), lower RT-PCR SARS-CoV-2 positivity rate (2.1% vs. 5.8%, *p <* 0.01), lower proportion of patients who had severe COVID-19 (0% vs. 0.5%, *p* = 0.79), lower proportion of patients who had symptomatic COVID-19 (1.8% vs. 6%, *p* = 0.22), and higher adverse effects rate (11.1% vs. 10.7%, *p* = 0.0066) than no treatment or other alternative treatment in the prevention of COVID-19. Conclusion: For PrEP, TGM/CGM-based treatment can be associated with a better clinical outcome than no treatment or other alternative treatment. However, more randomized control trials are warranted to confirm our findings and investigate the efficacy and safety of TGM/CGM to prevent COVID-19 in patients at risk for breakthrough or severe SARS-CoV-2 infection.

## 1. Introduction

**Figure 1:**
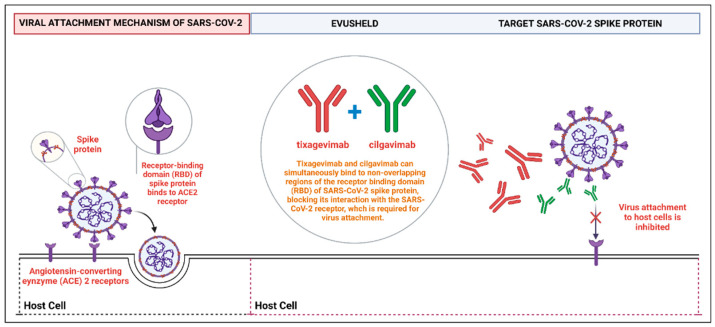
A schematic representation shows how TGM/CGM can simultaneously bind to the non-overlapping regions of the RBD of SARS-CoV-2 spike protein to prevent the virus attachment to human host cells. Abbreviations: RBD, receptor-binding domain; TGM/CGM, tixagevimab and cilgavimab; SARS-CoV-2, severe acute respiratory syndrome coronavirus 2.

Certain individuals may not benefit maximally from coronavirus disease 2019 (COVID-19) vaccines compared to the general population in whom COVID-19 vaccination is the optimal method of pre-exposure prophylaxis (PrEP) [1,2]. Severe acute respiratory syndrome coronavirus 2 (SARS-CoV-2) infection in people with moderate to severe immunocompromising conditions is associated with higher mortality compared to immunocompetent individuals, and immunocompromised individuals show an impaired anti-SARS-CoV-2 vaccine response even after four vaccine doses [3,4,5,6]. In this context, monoclonal antibody regimens (mAbs) providing passive immunization have been developed to enhance immunity against SARS-CoV-2 in immunocompromised patients [7]. In December 2021, tixagevimab-cilgavimab (TGM/CGM) received emergency use authorization (EUA) from the United States Food and Drug Administration (FDA) as PrEP in individuals aged 12 years or older (weighing at least 40 kg) who either are moderate to severely immunocompromised and cannot be vaccinated against COVID-19 or who may have an inadequate response to SARS-CoV-2 vaccination [7]. Subsequently, Europe approved TGM/CGM for PrEP in immunocompromised patients [8]. TGM/CGM is available as two separate injections given intramuscularly and given one after the other (Evusheld^®^, AstraZeneca Pharmaceuticals LP, Wilmington, DE 19850, USA), and the recommended revised dosing by the United States FDA is 600 mg, consisting of 300 mg of tixagevimab and 300 mg of cilgavimab to ensure the neutralization of this mAb combination against the COVID-19 Omicron subvariants, administered as separate sequential injections at different sites in two different muscles, preferably in the gluteal muscles [7]. TGM/CGM are neutralizing mAbs directed against different epitopes of the receptor-binding domain (RBD) of the SARS-CoV-2 spike protein and have been associated with a lower risk of SARS-CoV-2 infection when used for PrEP (See Figure 1).

The randomized controlled trial that led to authorization of the TGM/CGM combination involved 5197 adults (≥18 years or older) who were not vaccinated against SARS-CoV-2 and never transmitted COVID-19, and were deemed to be at high risk for breakthrough and severe SARS-CoV-2 infection (old age ≥ 60 years and/or many medical comorbidities) [9]. Overall, serious adverse events in this trial mentioned above were balanced between the TGM/CGM versus placebo groups [9]. Given the potential for TGM/CGM as an mAb therapy to prevent COVID-19, the combination has been evaluated in several small clinical cohorts [10,11,12]. Therefore, we carried out this meta-analysis to identify, describe, evaluate, and synthesize the evidence of efficacy and safety of TGM/CGM in patients at high risk for breakthrough and severe SARS-CoV-2 infection.

### Aim of the Study

This systematic review and meta-analysis aimed to assess the efficacy and safety of the monoclonal antibody combination TGM/CGM to prevent COVID-19 in patients at high risk for breakthrough and severe SARS-CoV-2 infection who might not benefit maximally from SARS-CoV-2 vaccination and for those who have a contraindication to SARS-CoV-2 vaccination in published research.

## 2. Methods

### 2.1. Design

This systematic review was conducted with reference to the basics of the *Cochrane Handbook for Systematic Reviews of Interventions* [13], described as stated by the Preferred Reporting Items for Systematic reviews and Meta-Analysis (PRISMA) statement [14].

### 2.2. Search Strategy and Selection Criteria

A systematic review protocol was developed based on PRISMA-P and the PRISMA statement. Published articles from 1 December 2021 to 30 November 2022, were selected for review from 8 electronic databases (PubMed, CINAHL, Embase, medRxiv, ProQuest, Wiley online library, Medline, and Nature). The focus of this review was the use of TGM/CGM to prevent COVID-19 in patients deemed to be at high risk for breakthrough and severe SARS-CoV-2 infection. The primary outcome was the efficacy of TGM/CGM in patients who might not benefit maximally from SARS-CoV-2 vaccination and for those who have a contraindication to SARS-CoV-2 vaccination. The secondary outcome was adverse events associated with its use.

### 2.3. Inclusion Criteria

Readily accessible peer-reviewed full articles, observational cohort studies, clinical trials, case reports, case series, and not peer-reviewed preprints were included.

### 2.4. Participants

Moderate to severe immunocompromised adult (aged ≥18 years) and child (aged ≥12 years) patients who cannot be vaccinated against COVID-19 or may have an inadequate response to SARS-CoV-2 vaccination were included.

### 2.5. Intervention

The interventions were the monoclonal antibody TGM/CGM, alone or in combination, versus an active comparator, placebo, or no intervention, for PrEP against COVID-19.

### 2.6. Study Variables

A. COVID-19-related hospitalization.

B. ICU admission.

C. Mortality.

D. Neutralization of COVID-19 Omicron variant.

E. Need of oxygen therapy.

F. RT-PCR SARS-CoV-2 positivity.

G. Severity of COVID-19.

H. Symptomatic COVID-19.

I. Safety and tolerability of TGM/CGM.

### 2.7. Searching Keywords

The search keywords included 2019-nCoV, 2019 novel coronavirus, COVID-19, coronavirus disease 2019, SARS-CoV-2, severe acute respiratory syndrome coronavirus 2, tixagevimab, cilgavimab, combination, monoclonal, passive, immunization, antibody, efficacy, clinical trial, cohort, pre-exposure, prophylaxis, and prevention.

### 2.8. Exclusion Criteria

Types of articles that were excluded included duplicate articles, editorials, reviews, and commentaries. Any research study that did not include data on TGM/CGM use, any study with in vitro or in silico activity of TGM/CGM to prevent SARS-CoV-2 variants, any study with no relevant data, or any study with no extractable data was also excluded. We excluded studies on the use of monoclonal antibody TGM/CGM to treat COVID-19, as this is not part of the aim of our review.

### 2.9. Data Extraction and Analysis

The screening of the papers was performed independently by four reviewers (Saad Alhumaid, Jalal Alali, Nourah Al Dossary, and Sami Hussain Albattat), by screening titles with abstracts using the selection criteria. Disagreements in the study selection after the full text screening were discussed, and if agreement could not be reached, a fifth reviewer was involved (Sarah Mahmoud Al HajjiMohammed). We categorized articles as case reports, case series, clinical trials, or cohort studies. The following data were extracted from the selected studies: authors; publication year; study location; study design and setting; age, gender, and sample size; details of study intervention and control therapies in addition to data on adverse events and treatment outcomes; time from TGM/CGM administration to first episode of symptomatic COVID-19; assessment of study risk of bias; and remarks on notable findings.

### 2.10. Risk of Biased Evaluation of Included Studies

Three tools were used appropriately to assess the quality of the studies included in this review: (1) Newcastle–Ottawa Scale (NOS) to evaluate cohort studies (scoring criteria: >7 scores = high quality, 5–7 scores = moderate quality, and <5 scores = low quality) [15]; (2) modified NOS to evaluate case report and case series studies (scoring criteria: 5 criteria fulfilled = good, 4 criteria fulfilled = moderate, and 3 criteria fulfilled = low) [16]; and (3) Revised Cochrane risk-of-bias tool (RoB 2.0) to evaluate randomized controlled trials (bias is assessed in five distinct domains and answers lead to judgments of “low risk of bias,” “some concerns,” or “high risk of bias”) [17]. Quality assessment was conducted by three co-authors (Fatimah Saad Almuaiweed, Sukainah Mohammad Alshaikhnasir, and Maryam Radhi AlZaid) who separately evaluated the possibility of bias using these three tools.

### 2.11. Assessment of Heterogeneity

For clinical outcomes, safety and tolerability parameters of TGM/CGM mAbs, Cochran’s test for chi-squared (*χ*^2^) expressed as the Higgins (*I*^2^) were used to measure the statistical heterogeneity [13]. Degrees of heterogeneity were categorized based on calculated *I*^2^ values (not significant: 0–<40%; moderate: 30–60%; substantial: 50–90%; and significant: 75–100%).

### 2.12. Statistical Analysis

Because all of the data were continuous and dichotomous data, risk ratios (RRs) were used for estimating the point estimate, along with 95% confidence intervals (CIs). Taking a conservative approach, a random effects with the DerSimonian–Laird model was used [18], which produces wider CIs than a fixed effect model. Publication bias was evaluated using funnel plots and the Egger’s correlation test, with *p* < 0.05 indicating statistical significance [19]. R version 4.1.0 with the packages *metafor* and *meta* was used to conduct all statistical analyses and generate forest plots. Figure 1 was created with BioRender.com (accessed on 11 August 2022) (agreement no. PW249QJYDR).

## 3. Results

### 3.1. Study Characteristics and Quality

**Figure 2:**
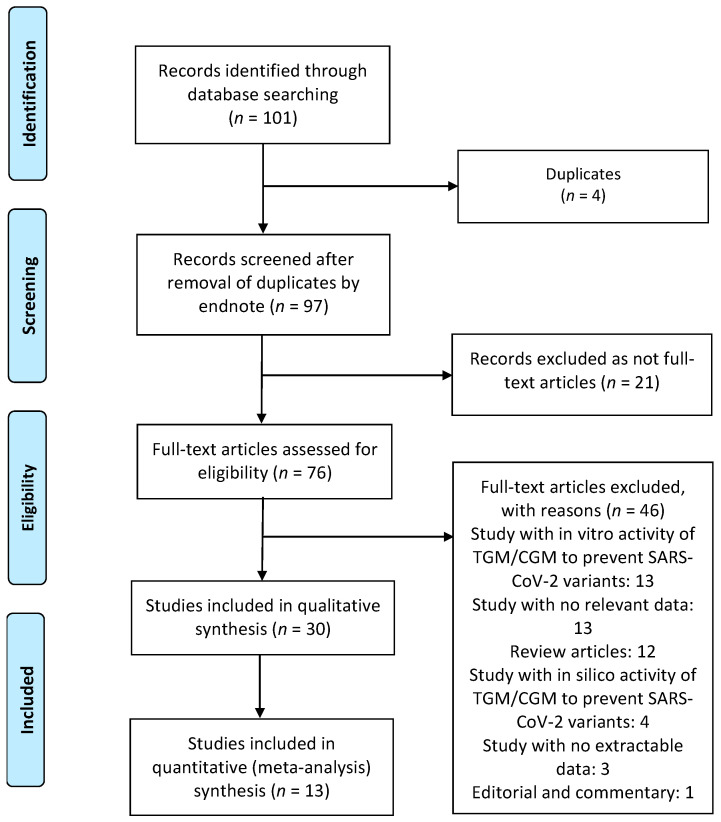
Preferred Reporting Items for Systematic reviews and Meta-Analysis (PRISMA) flow chart of the included studies. SARS-CoV-2, severe acute respiratory syndrome coronavirus 2; TGM/CGM, tixagevimab/cilgavimab.

**Figure 3:**
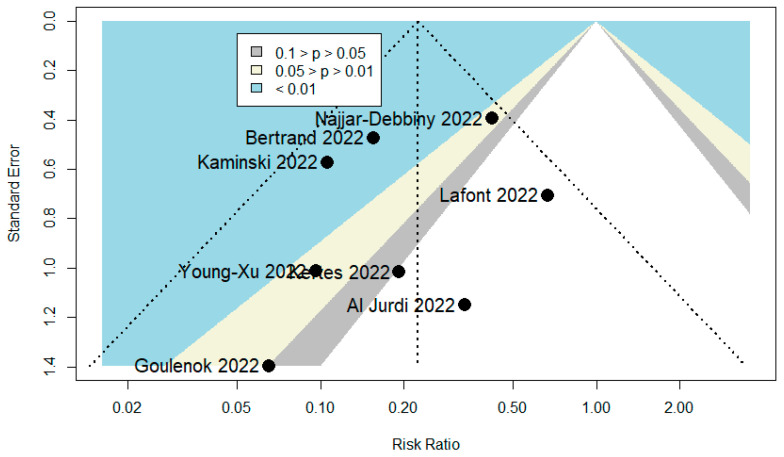
Funnel plot to evaluate publication bias for the pooled effect size to determine the rate of COVID-19-related hospitalization associated with TGM/CGM-based treatment in patients at high risk for breakthrough and severe SARS-CoV-2 infection.

**Figure 4:**
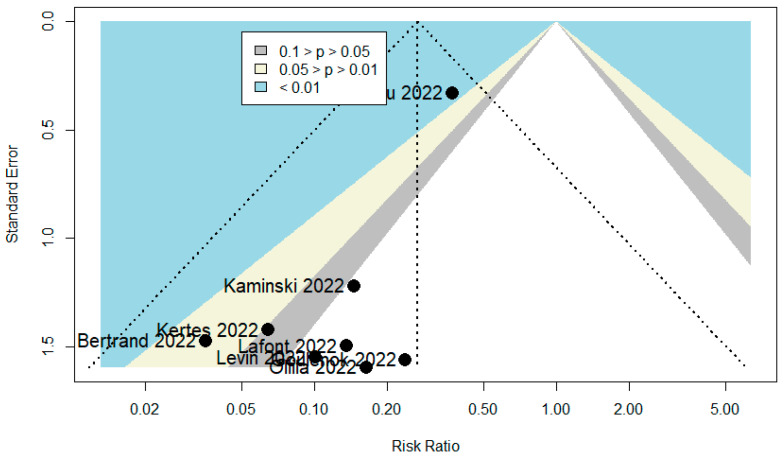
Funnel plot to evaluate publication bias for the pooled effect size to determine the rate of mortality associated with TGM/CGM-based treatment in patients at high risk for breakthrough and severe SARS-CoV-2 infection.

**Figure 5:**
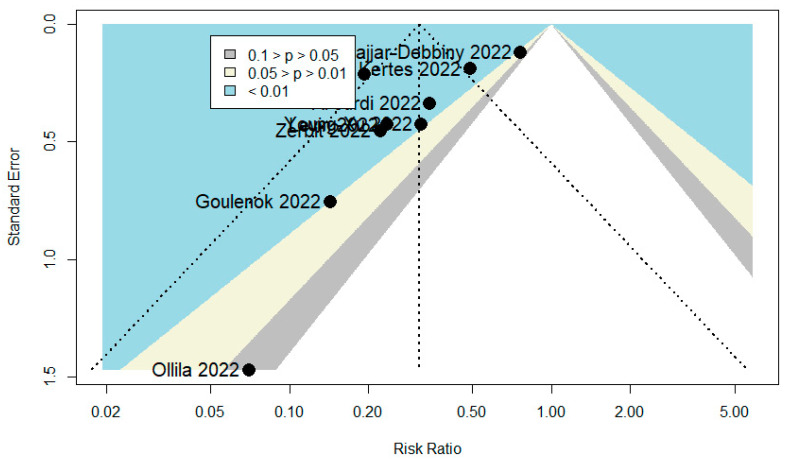
Funnel plot to evaluate publication bias for the pooled effect size to determine the rate of RT-PCR positivity for SARS-CoV-2 associated with TGM/CGM-based treatment in patients at high risk for breakthrough and severe SARS-CoV-2 infection.

A total of 101 publications were identified. After scanning titles and abstracts, four duplicate articles were discarded. Another 21 irrelevant articles were excluded based on the titles and abstracts. The full texts of the 76 remaining articles were reviewed, and 46 irrelevant articles were excluded (reasons: study with in vitro activity of TGM/CGM to prevent SARS-CoV-2 variants = 13, study with no relevant data = 13, review = 12, study with in silico activity of TGM/CGM to prevent SARS-CoV-2 variants = 4, study with no extractable data = 3, and editorial and commentary = 1). As a result, we identified 30 studies (total participants = 27,932) that met our inclusion criteria and were included in the systematic review and meta-analysis [6,9,10,11,12,20,21,22,23,24,25,26,27,28,29,30,31,32,33,34,35,36,37,38,39,40,41,42,43,44]. The PRISMA chart for the studies included is displayed in Figure 2. The details of the included studies are depicted in Table 1. Among these, two articles were in preprint versions [26,35]. Most of the studies were cohorts (*n* = 23) [6,10,11,12,20,21,22,23,26,27,30,31,33,35,36,37,38,39,40,41,42,43,44], however, there were one case report [24] and five case series studies [25,28,29,32,34]. These studies were conducted in United States (*n* = 14), France (*n* = 11), Israel (*n* = 2), Germany (*n* = 1), and Austria (*n* = 1). Only one study was conducted within several countries (*n* = 1) [9]. The majority of the studies were single center [6,11,12,23,24,25,26,28,29,31,32,33,34,36,37,38,39,41,42,44], and only ten studies were multicenter [9,10,20,21,22,27,30,35,40,43]. Studies randomly assigned participants into a TGM/CGM therapy versus a placebo [9,10,12,35], a combination of casirivimab/imdevimab (CRM/IDM) [11,21,37], or no treatment (no mAbs) [11,25,27,29,31,37,40]. Few studies utilized a pre–post design (baseline) [6,23,26,36] and only one study utilized the design of randomized double-blind controlled trial [9]. Many studies compared TGM/CGM groups to no comparator groups [20,22,24,28,30,32,33,34,36,38,39,41,42,43,44] or patient groups that were infected previously with SARS-CoV-2 and recovered [21]. Only twelve studies reported on the safety of TGM/CGM in patients at high risk for breakthrough and severe SARS-CoV-2 infection [9,10,11,25,26,28,34,39,41,42,43,44]. Among the 30 included studies, 14 studies were moderate-quality studies (i.e., NOS scores were between 5 and 7 or based on the assessment for bias using the modified NOS) and 15 studies demonstrated relatively high quality (i.e., NOS scores > 7 or based on the assessment for bias using the modified NOS). The only study that utilized the design of randomized double-blind controlled trial had a low risk of bias based on RoB 2 (Table 1). The funnel plots for possible publication bias for the pooled effect sizes to determine the rates of COVID-19-related hospitalization, mortality, and RT-PCR positivity for SARS-CoV-2 associated with the TGM/CGM-based monoclonal preventive therapy in patients at high risk for breakthrough and severe SARS-CoV-2 infection appeared asymmetrical on visual inspection, and Egger’s tests confirmed symmetry by producing *p* values < 0.05 (Figure 3, Figure 4 and Figure 5).

### 3.2. Demographic and Clinical Characteristics of Patients in the TGM/CGM and Control Groups

The included studies had a total of 27,932 patients (11,720 participants were in the TGM/CGM groups, and 16,212 participants were in the control therapy groups) who were deemed to be at high risk for breakthrough and severe SARS-CoV-2 infection, as detailed in Table 1. Amongst these studies, all reported TGM/CGM combination use in adult patients (100%). The patients’ ages ranged from 19.7 years to 79.8 years across studies. There was a slightly higher male percentage in patients who received TGM/CGM as PrEP against COVID-19 (*n* = 5590/8903, 62.8%). Participants were immunocompromised (*n* = 18,611) [22,25,27,29,30,32,35,39,40,44], high-risk patients for severe COVID-19 (obesity (BMI ≥ 30), hypertension, smoking, diabetes, asthma, chronic heart or kidney or liver diseases, or receipt of immunosuppressive therapy) (*n* = 8621) [9,30,35,44], solid organ transplant recipients (kidney, liver, lung, and multiorgan) (*n* = 3111) [6,10,11,12,20,21,26,28,30,38,42,44], hematologic malignancy patients (*n* = 1320) [30,31,32,33,37,38,44], inborn error of immunity and immune-mediated inflammatory diseases patients (*n* = 445) [30,38,43], B-cell malignancy patients (*n* = 251) [41], autoimmune disease patients (*n* = 229) [25,32,38,44], multiple sclerosis patients (*n* = 45) [23,34,38], anti-neutrophil cytoplasmic antibody-associated vasculitis patients (*n* = 34) [36,39], or rheumatoid arthritis patients (*n* = 28) [22,39]. Most of the patients received an intramuscular TGM/CGM 150–150 mg dose (*n* = 10,503) [6,9,11,12,20,21,23,24,25,27,28,29,30,31,32,33,34,35,37,38,39,40,41,42,43], however, some participants received an intramuscular TGM/CGM 300–300 mg dose (*n* = 1198) [10,22,26,36,39,41,42,43,44], and only one individual received the 450–450 mg dose (*n* = 1) [10]. The median interquartile range (IQR) time from TGM/CGM administration to the first episode of symptomatic COVID-19 was 18 (9–32) days. Among the studies that included comparator groups to TGM/CGM [9,10,11,12,21,25,27,29,31,35,37,40], age, sex, comorbidities, and other baseline characteristics were generally balanced between both the TGM/CGM and control groups in all studies except for one study [21]. There was a tendency for a higher frequency of comorbidities in the control arms (however, this was not significant) [12,25,27,29]; furthermore, the number of doses of COVID-19 vaccine or/and proportion of patients who received CRM/IDM as a first-step protection and the proportion of patients with prior SARS-CoV-2 infection were not balanced [10,11,12,27,29,35,37].

### 3.3. TGM/CGM and Outcomes

Based on the findings of this study, TGM/CGM-based treatment was associated with a lower COVID-19-related hospitalization rate (0.54% vs. 1.2%, *p* = 0.27), lower ICU admission rate (0.6% vs. 5.2%, *p* = 0.68), lower mortality rate (0.2% vs. 1.2%, *p* = 0.67), higher neutralization of COVID-19 Omicron variant rate (12.9% vs. 6%, *p* = 0.60), lower proportion of patients who needed oxygen therapy (8% vs. 41.2%, *p* = 0.27), lower RT-PCR SARS-CoV-2 positivity rate (2.1% vs. 5.8%, *p <* 0.01), lower proportion of patients who had severe COVID-19 (0% vs. 0.5%, *p* = 0.79), lower proportion of patients who had symptomatic COVID-19 (1.8% vs. 6%, *p* = 0.22), and higher adverse effects rate (11.1% vs. 10.7%, *p* = 0.0066) than no treatment or other alternative treatment in the prevention of COVID-19. In summary, our findings indicate that TGM/CGM can be a potential therapeutic agent to prevent COVID-19 in patients at high risk for breakthrough and severe SARS-CoV-2 infection.

### 3.4. Clinical Outcomes

#### 3.4.1. COVID-19-Related Hospitalization

**Figure 6:**
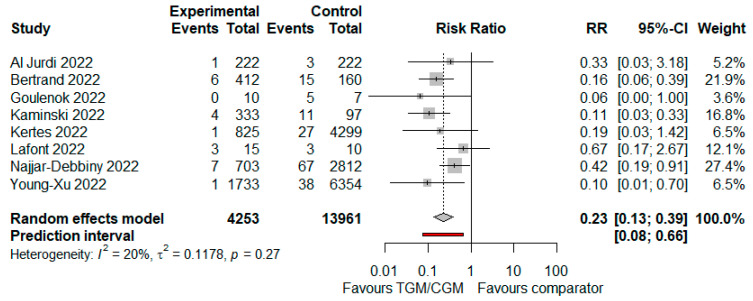
Rate of COVID-19-related hospitalization (TGM/CGM vs. comparator). CI, confidence interval; RR, risk ratio; TGM/CGM, tixagevimab/cilgavimab.

In the pooled analysis of seven studies, the COVID-19-related hospitalization rate in the TGM/CGM-based treatment was lower than that of the control group (COVID-19-related hospitalization rate: 23/4253 (0.54%) vs. 169/13,961 (1.2%), RR = 0.23; 95% CI, 0.13 to 0.39; *I*^2^ = 20%, *p* = 0.27, Figure 6) [10,11,12,25,27,29,35,40].

#### 3.4.2. ICU Admission

**Figure 7:**
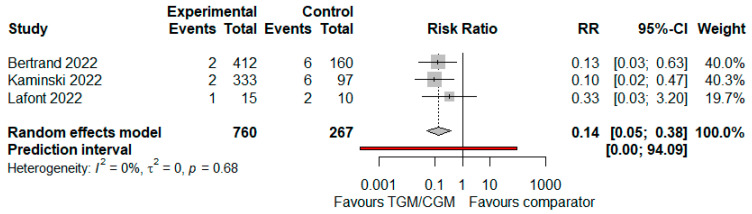
Rate of ICU admission (TGM/CGM vs. comparator). CI, confidence interval; RR, risk ratio; TGM/CGM, tixagevimab/cilgavimab.

In the pooled analysis of three studies, ICU admission in the TGM/CGM-based treatment was lower than that of the control group (mortality rate: 5/760 (0.6%) vs. 14/267 (5.2%), RR = 0.14; 95% CI, 0.05 to 0.38; *I*^2^ = 0%, *p* = 0.68, Figure 7) [11,12,29].

#### 3.4.3. Mortality

**Figure 8:**
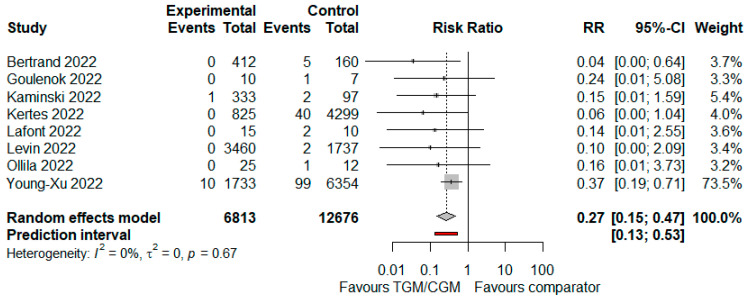
Rate of mortality (TGM/CGM vs. comparator). CI, confidence interval; RR, risk ratio; TGM/CGM, tixagevimab/cilgavimab.

In the pooled analysis of eight studies, mortality rate in the TGM/CGM-based treatment was lower than that of the control group (mortality rate: 11/6813 (0.2%) vs. 152/12,676 (1.2%), RR = 0.27; 95% CI, 0.15 to 0.47; *I*^2^ = 0%, *p* = 0.67, Figure 8) [9,11,12,25,27,29,31,35].

#### 3.4.4. Neutralization of COVID-19 Omicron Variant

**Figure 9:**
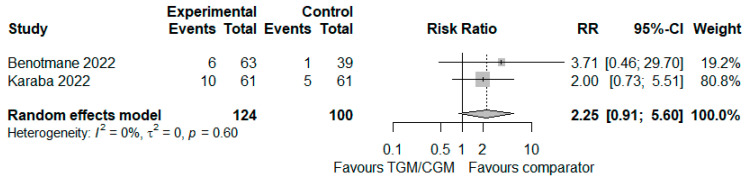
Rate of neutralization of COVID-19 Omicron variant (TGM/CGM vs. comparator). CI, confidence interval; RR, risk ratio; TGM/CGM, tixagevimab/cilgavimab.

In the pooled analysis of two studies, the rate of patients with neutralization of the COVID-19 Omicron variant in the TGM/CGM-based treatment was higher than that of the control group (rate of neutralization of COVID-19 Omicron variant: 16/124 (12.9%) vs. 6/100 (6%), RR = 2.25; 95% CI, 0.91 to 5.60; *I*^2^ = 0%, *p* = 0.60, Figure 9) [21,26].

#### 3.4.5. Need for Oxygen Therapy

**Figure 10:**
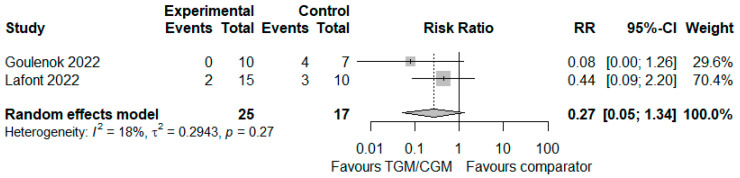
Proportion of patients who needed oxygen therapy (TGM/CGM vs. comparator). CI, confidence interval; RR, risk ratio; TGM/CGM, tixagevimab/cilgavimab.

In the pooled analysis of two studies, the proportion of patients who needed oxygen therapy in the TGM/CGM-based treatment was lower than that of the control group (proportion of patients who needed oxygen therapy: 2/25 (8%) vs. 7/17 (41.2%), RR = 0.27; 95% CI, 0.05 to 1.34; *I*^2^ = 0%, *p* = 0.27, Figure 10) [25,29].

#### 3.4.6. RT-PCR SARS-CoV-2 Positivity

**Figure 11:**
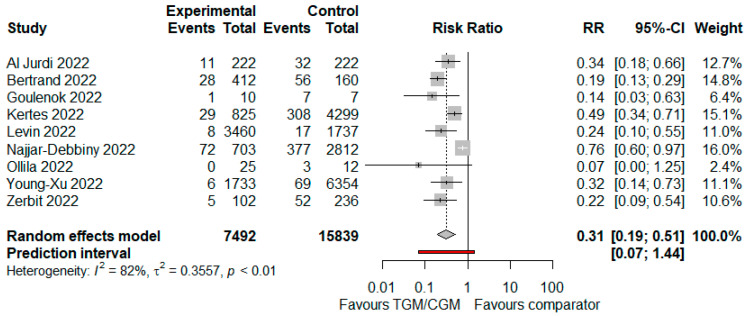
Rate of RT PCR SARS-CoV-2 positivity (TGM/CGM vs. comparator). CI, confidence interval; RR, risk ratio; TGM/CGM, tixagevimab/cilgavimab.

In the pooled analysis of seven studies, the RT PCR SARS-CoV-2 positivity rate in the TGM/CGM-based treatment was significantly lower than that of the control group (RT PCR SARS-CoV-2 positivity rate: 160/7492 (2.1%) vs. 921/15,839 (5.8%), RR = 0.31; 95% CI, 0.19 to 0.51; *I*^2^ = 82%, *p <* 0.01, Figure 11) [9,10,11,25,27,31,35,37,40].

#### 3.4.7. Severity of COVID-19

**Figure 12:**
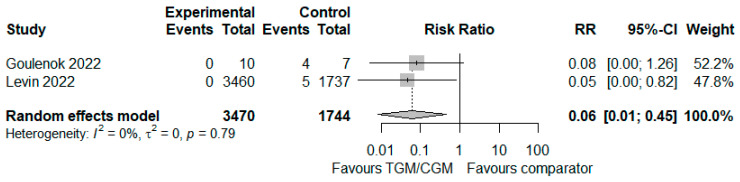
Proportion of patients who experienced severe COVID-19 (TGM/CGM vs. comparator). CI, confidence interval; RR, risk ratio; TGM/CGM, tixagevimab/cilgavimab.

In the pooled analysis of two studies, the proportion of patients who experienced severe COVID-19 in the TGM/CGM-based treatment was lower than that of the control group (proportion of patients who experienced severe COVID-19: 0/3470 (0%) vs. 9/1744 (0.5%), RR = 0.06; 95% CI, 0.01 to 0.45; *I*^2^ = 0%, *p* = 0.79, Figure 12) [9,25].

#### 3.4.8. Symptomatic COVID-19

**Figure 13:**
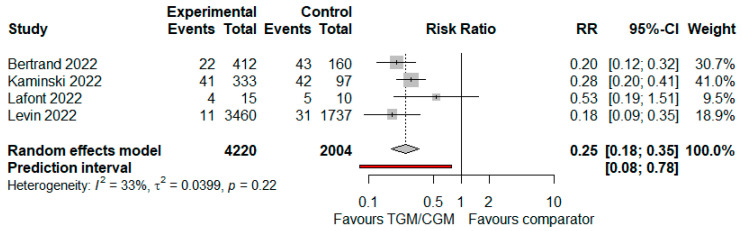
Proportion of patients who had symptomatic COVID-19 (TGM/CGM vs. comparator). CI, confidence interval; RR, risk ratio; TGM/CGM, tixagevimab/cilgavimab.

In the pooled analysis of four studies, the proportion of patients who had symptomatic COVID-19 in the TGM/CGM-based treatment was lower than that of the control group (proportion of patients who had symptomatic COVID-19: 78/4220 (1.8%) vs. 121/2004 (6%), RR = 0.25; 95% CI, 0.18 to 0.35; *I*^2^ = 33%, *p* = 0.22, Figure 13) [9,11,12,29].

### 3.5. Safety and Tolerability

**Figure 14:**
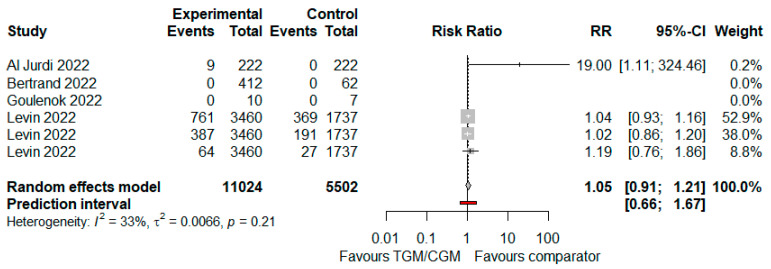
Rate of adverse effects (TGM/CGM vs. comparator). CI, confidence interval; RR, risk ratio; TGM/CGM, tixagevimab/cilgavimab.

In the pooled analysis of four studies, the adverse effects rate in the TGM/CGM-based treatment was significantly higher than that of the control group (adverse effects rate: 1221/11,024 (11.1%) vs. 587/5502 (10.7%), RR = 1.05; 95% CI, 0.91 to 1.21; *I*^2^ = 33%, *p* = 0.0066, Figure 14) [9,10,11,25].

## 4. Discussion

This is the largest meta-analysis on the efficacy and safety of TGM/CGM as mAb combination in patients at high risk for breakthrough and severe SARS-CoV-2 infection. This study involving 27,932 participants from 23 cohort, 5 case series, 1 case report, and 1 randomized clinical trial studies found that the majority of the patients treated with TGM/CGM as PrEP against COVID-19 were adults (100%), men (62.8%), and were mainly immunocompromised (66.6%) or high-risk patients for severe COVID-19 (e.g., obesity (BMI ≥ 30), hypertension, smoking, diabetes, or asthma (30.9%)). Our meta-analysis showed that the use of the TGM/CGM-based mAb combination in patients at high risk for breakthrough and severe SARS-CoV-2 infection significantly lowered the RT-PCR SARS-CoV-2 positivity rate and was associated with higher adverse effects (*p* < 0.05). The overall rates of COVID-19-related hospitalization, ICU admission, mortality, neutralization of COVID-19 Omicron variant, need for oxygen therapy, and severe and symptomatic COVID-19 were in favor of TGM/CGM regimens compared to an active comparator, placebo, or no intervention for PrEP against COVID-19. The findings of this review should be interpreted carefully. We included 12 studies with small sample sizes which were conducted exclusively in two countries only (France and the United States) [6,20,22,23,24,25,26,28,29,31,33,39], an issue that may limit the generalizability of our findings. Nevertheless, the heterogeneity regarding the clinical outcomes among 4 out of these 12 studies when combined with the other included studies was small, which could limit the bias in this meta-analysis [25,26,29,31].

Monoclonal antibodies are considered potential nonvaccine drugs to provide rapid protection against COVID-19 regardless of the recipient’s immune system status [45,46]. The TGM/CGM mAb combination targeting the RBD of the SARS-CoV-2 spike protein is modified in the Fc regions to improve the half-life and decrease Fc effector functions [47]. If the results from this meta-analysis were confirmed in larger randomized trials, TGM/CGM would be an important preventive option against SARS-CoV-2. The injectable dosing, established safety profile, acceptable costs of production, and large-scale manufacture of TGM/CGM could allow rapid expansion to worldwide use in the prevention of COVID-19. However, the prophylactic efficacy of TGM/CGM is challenged by emerging immune-evasive SARS-CoV-2 variants [6,21,22,26,33,41,42], notably since the COVID-19 Omicron subvariants have emerged and become dominant worldwide with characteristics that allow them to evade PrEP from the mAb combination [48]. TGM/CGM is active against Omicron (B.1.1.529) lineage variants, but activity may be reduced depending on the sublineage (e.g., 12- to 30-fold decrease in susceptibility for BA.1 and 176-fold decrease for BA.1.1 [49], minimal change in susceptibility for BA.2 (5.4-fold decrease), and likely active (moderate reduction in susceptibility) for sublineages BA.4 and BA.5 [50,51]). With the relaxing of infection prevention and control measures across most countries, it is vital to protect patients at highest risk of serious COVID-19, whereas rates of Omicron subvariants’ infection are still high. Immunocompromised patients are fatigued themselves to keep socially isolating, physically distancing, and avoiding others. Therefore, the use of TGM/CGM in these susceptible individuals may be a valued strategy to provide better protection against serious consequences from COVID-19. We should emphasize the need for additional prevention measures in people at high risk for breakthrough or severe COVID-19, such as masking and completing immunization series, while SARS-CoV-2 transmission remains high in the community. PrEP-reduced effectiveness of TGM/CGM justified the FDA’s subsequent action to revise the TGM/CGM regimen and supports the necessity to give the TGM/CGM mAb combination at a dose higher than 150 mg/150 mg to ensure the neutralization of TGM/CGM against SARS-CoV-2 Omicron and its subvariants as variants of concern. However, there is a potential to intoxicate the patient, because high doses of TGM/CGM might have led to the very few reported cases of myocardial infarction, mild heart failure pericarditis, and mild/moderate cardia allograft rejection (*n* = 4) [9,10,42]. In a recent population-based propensity-matched cohort study, TGM/CGM use for PrEP against COVID-19 was not associated with increased risk of myocardial infarction, arrhythmias, or heart failure [52]. A causal relationship between TGM/CGM and these reported cardiovascular events has not been established. Risks and benefits before initiating TGM/CGM in individuals at high risk for cardiovascular events should be considered, and patients should be advised to seek immediate medical attention if they experience any signs or symptoms suggestive of a cardiovascular event. Hence, COVID-19 itself is strongly associated with arterial and venous complications [53,54,55,56], TGM/CGM can be hypothesized to lower the risk for myocardial infarction through the prevention of SARS-CoV-2 infection in select individuals at high risk of progression to severe COVID-19. Although evidence on the cost effectiveness of the use of TGM/CGM within its marketing authorization for preventing COVID-19 compared with the current standard of care is lacking, use of TGM/CGM mAbs in moderately to severely compromised patients as a PrEP may be cost effective by preventing severe outcomes and reducing hospitalization, thereby allowing patients a more rapid return to work. The use of TGM/CGM in suitable patients may improve quality of life by allowing patients to return to daily living activities with more confidence that include increased normal social interaction with others.

Currently, there is only one published meta-analysis that addressed the efficacy and safety of TGM/CGM as PrEP therapy in participants at high risk for breakthrough and severe SARS-CoV-2 infection [57]. Regarding the occurrence of adverse effects in patients who received TGM/CGM-based treatment, we report an overall similar adverse effects rate compared to the previous meta-analysis (RR: 1.05 vs. 1.00) [57]. However, we report a higher pooled effect size of COVID-19-related hospitalization (RR: 0.23 vs. 0.03) and symptomatic COVID-19 (RR: 0.25 vs. 0.18), but a lower pooled effect size of mortality (RR: 0.27 vs. 0.64) and RT-PCR SARS-CoV-2 positivity (RR: 0.31 vs. 0.45) compared to the previous meta-analysis [57]. Our meta-analysis is current and more comprehensive and included a total of 30 studies [6,9,10,11,12,20,21,22,23,24,25,26,27,28,29,30,31,32,33,34,35,36,37,38,39,40,41,42,43,44] including a total of 27,932 participants whose details on final treatment outcome were available, in comparison to a smaller sample size in the previous meta-analysis (*n* = 5197) that was based on the outcomes evidence of a single study [9]. The inclusion of 28 recently published studies [6,10,11,12,20,21,22,23,24,25,26,27,28,29,30,31,32,33,34,35,37,38,39,40,41,42,43,44] contributed to the refinement on evidence of the demographic, laboratory, and clinical characteristics, in addition to final therapy outcomes and safety concerns in patients at high risk for breakthrough and severe SARS-CoV-2 infection treated with the preventive TGM/CGM.

In terms of safety, this study found greater adverse events reported in the TGM/CGM-based treatment versus control groups [9,10,11,25,39,41,42,43]. Most adverse events associated with TGM/CGM alone or in combination with other medicines were reported in solid organ recipients [10,28,42], and were typically gastrointestinal disorders (including nausea, vomiting, diarrhea, abdominal pain (*n* = 8)) [10,28,41]; nevertheless, a few serious adverse drug reactions such as mild heart failure, new atrial fibrillation requiring cardioversion, pericarditis, mild/moderate cardiac allograft rejection, or renal failure (*n* = 5) were also reported [9,10,42]. A few cases of myalgia (*n* = 6) and fatigue (*n* = 2) were also reported [28,39]. There have been no reported cases for serious or severe adverse effects attributed to the study medications or necessitating interruption of treatment.

### Limitation of the Study

We acknowledge that our study was not without some limitations. First, all of the evidence discussed was based on many cohorts and a few case series studies which were small in sample size, and most were performed within single centers. Second, most studies included in this review were retrospective in design which could have introduced potential reporting bias due to reliance on clinical case records. Third, we included two non-peer-reviewed studies, and as such their effect on the final pooled treatment outcomes may be confounded. Fourth, the study population included adult patients only, and hence its results cannot be generalized to pediatric patients. Finally, the study was not registered in Prospero, an international prospective register of systematic reviews, as this might have added extra work and the merit was mostly limited to the avoidance of duplication.

## 5. Conclusions

For PrEP, there is a decrease in the development of COVID-19 related hospitalization, ICU admission, mortality, need for oxygen therapy, RT-PCR SARS-CoV-2 positivity, and severity and symptoms of COVID-19; however, there is a higher rate for all adverse effects with TGM/CGM. A higher rate of neutralization of COVID-19 Omicron subvariants was found with TGM/CGM use. TGM/CGM is active against Omicron (B.1.1.529) lineage variants but its activity may be reduced for BA.1, BA.1.,1 and BA.2, and likely maintains most of its activity against activity for sublineages BA.4 and BA.5. TGM/CGM higher than a 300 mg/300 mg dose may ensure more neutralization against SARS-CoV-2 Omicron and its subvariants in comparison to the TGM/CGM 150 mg/150 mg dose in immunocompromised patients; however, there is a higher potential to intoxicate the patient, particularly solid organ transplant recipients. More randomized control trials are warranted to confirm our findings and investigate the efficacy and safety of TGM/CGM to prevent COVID-19 in patients at risk for breakthrough or severe SARS-CoV-2 infection.

## Figures and Tables

**Table 1:** Data extracted from included papers (*n* = 30).

Author, Year [Reference], Study Location	Study Design and Setting	Age (Year)	Male, *n* (%)	Population	Intervention	Control	Time from TGM/CGM Administration to First Episode of Symptomatic COVID-19 (Days)	Outcome	AEs in TGM/CGM and Control Arm	Assessment of Study Risk of Bias (Tool Used; Finding)	Remark
Al Jurdi et al., 2022 [10], United States	Retrospective cohort; multicenter	Median (IQR), 65 (55–72)	136 (61.3)	SOTRs	222 SOTRs received TGM/CGM (IM) TGM/CGM (IM) (150–150 mg dose) (*n* = 90), TGM/CGM (IM) (300–300 mg dose) (*n* = 131), and TGM/CGM (IM) (450–450 mg dose) (*n* = 1)	222 SOTRs received placebo	Median (IQR), 81 (15–97)	***RT-PCR positivity for SARS-CoV-2:*** positive PCR for SARS-CoV-2 was lower in the TGM/CGM group vs. placebo (11 vs. 32, *p* < 0.001). SARS-CoV-2 infection was higher in those who received the lower (150–150 mg) dose of TGM/CGM compared to those who received the higher dose of 300–300 mg (*p* = 0.025). ***COVID-19-related hospitalization:*** percentage of patients admitted to hospital was lower among TGM/CGM group vs. placebo group (1 vs. 3, *p* > 0.05). *Mortality:* 0 patients allocated to TGM/CGM and 3 patients allocated to placebo died (*p* > 0.05).	AEs in the TGM/CGM group occurred in 9 SOTRs. Most common AEs were nausea, vomiting, or diarrhea (*n* = 4), headache (*n* = 3), and abdominal pain (*n* = 2). Two patients developed new lung infiltrates with negative infectious evaluation, thought to be pneumonitis. One patient developed a mild heart failure exacerbation, and one developed new atrial fibrillation.	NOS, 8	Control group had similar characteristics to the TGM/CGM group.
Al-Obaidi et al., 2022 [44], United States	Retrospective cohort; single center	Median (IQR), 68 (58–75)	238 (51.4)	ICHs	463 ICH participants received TGM/CGM (IM) (300–300 mg dose)	No comparator group	Median (IQR), 48 (27.5–69)	***RT-PCR positivity for SARS-CoV-2:*** 6/98 developed COVID-19. ***COVID-19-related hospitalization:*** 42/463 required hospitalization. ***Mortality****:* 0 patients died attributed to COVID-19.	No severe AEs were recorded for TGM/CGM in all patients.	NOS, 8	All patients meeting the criteria for therapy did not have a recent exposure or an acute COVID-19 infection.
Aqeel et al., 2022 [36], United States	Retrospective cohort; single center	Mean (SD), 66 (15.5)	Not reported	AAV patients	20 AVV participants received TGM/CGM (IM) (300–300 mg dose)	No comparator group	114, 57, and 125	***RT-PCR positivity for SARS-CoV-2:*** 3/20 developed COVID-19. ***COVID-19-related hospitalization:*** 0 patients required hospitalization. ***Severity of COVID-19:*** 3 cases were classified as mild disease.	Not reported	NOS, 6	All patients received the COVID-19 vaccine.
Benotmane et al., 2022 [20], France	Retrospective cohort; multicenter	Median (IQR), 60.1 (52.3–71.9)	23/39 (59)	KTRs	416 SOTRs received TGM/CGM (IM) (150–150 mg dose)	No comparator group	Median (IQR), 20 (9.5–34.5)	***RT-PCR positivity for SARS-CoV-2:*** 39 patients developed COVID-19. ***COVID-19-related hospitalization:*** 14 patients required hospitalization. ***ICU admission:*** 3 patients were transferred to ICU. ***Mortality:*** 2 patients died of COVID-19-related ARDS.	Not reported	NOS, 6	Patients who had already received the CRM-IDM combination were not excluded (*n* = 25).
Benotmane et al., 2022 [21], France	Retrospective cohort; multicenter	Not reported	Not reported	KTRs	63 KTRs received TGM/CGM (IM) (150–150 mg dose)	39 KTRs received CRM/IDM (IV) (600–600 mg dose) and 14 KTRs were infected with SARS-CoV-2	Median (IQR), 29 (29–33)	***Neutralization of COVID-19 Omicron variant:*** 6/63 (TGM/CGM), 1/39 (CRM/IDM), and 10/14 (patients who were infected with COVID-19) were able to neutralize Omicron.	Not reported	NOS, 6	High interindividual variability in the formed neutralizing antibodies was observed in the TGM/CGM group, which was explained largely by the patients’ BMI.
Benotmane et al., 2022 [6], France	Retrospective cohort; single center	Median (IQR), 55.5 (50–67.8)	53 (54)	KTRs	98 KTRs received TGM/CGM (IM) (150–150 mg dose)	Same KTR patients before receiving TGM/CGM (at baseline)	Not reported	***Neutralization of COVID-19 Omicron variant:*** neutralizing activity decreased from 2.7 log_10_ to 2.4 log_10_ between 1 month and month 4–5 following TGM/CGM injection, *p* = 0.007.	Not reported	NOS, 7	Seventy-two patients had been previously treated with the CRM/IDM combination before the emergence of the Omicron variant.
Bertrand et al., 2022 [11], France	Retrospective cohort; single center	Mean (SD), 60.2 (14.2)	254 (61.6)	KTRs	412 KTRs received TGM/CGM (IM) (150–150 mg dose)	62 KTRs received CRM/IDM (IV) (600–600 mg dose) and 98 KTRs received no mAbs	Not reported	***RT-PCR positivity for SARS-CoV-2:*** positive PCR for SARS-CoV-2 was lower in the TGM/CGM group vs. CRM/IDM and no mAbs group (28 vs. 56, *p* < 0.001). ***Symptomatic COVID-19:*** 22 developed symptomatic COVID-19 in the TGM/CGM group compared to 43 among those who received CRM/IDM or no mAbs (*p* < 0.001). ***COVID-19-related hospitalization:*** percentage of patients admitted to hospital was lower among TGM/CGM group vs. CRM/IDM or no mAbs group (6 vs. 15, *p* < 0.001). ***ICU admission:*** lower number of patients in the TGM/CGM group required ICU admission compared to patients in the CRM/IDM or no mAbs group (2 vs. 6, *p* = 0.0009). ***Mortality:*** 0 patients allocated to TGM/CGM and 5 patients allocated to CRM/IDM or no mAbs died, *p* = 0.0026.	No severe AEs were recorded for TGM/CGM in all patients.	NOS, 7	Most patients received CRM/IDM as a 1st step protection (*n* = 267).
Bruel et al., 2022 [22], France	Prospective cohort; multicenter	Median (IQR), 61 (31–92)	15 (52)	Immunocompromised patients (RA, kidney graft, vasculitis, polychondritis, and SLE)	29 immunocompromised patients received TGM/CGM (IM) (300–300 mg dose)	No comparator group	Median (IQR), 18 (12.7–22.5)	***RT-PCR positivity for SARS-CoV-2:*** 4 patients developed COVID-19 (all cases were Omicron). ***Severity of COVID-19:*** 3 cases were classified as mild disease, whereas 1 case was classified as severe. ***COVID-19-related hospitalization:*** 1 patient required hospitalization.	Not reported	NOS, 7	Most patients (*n* = 18) were previously treated with CRM/IDM before TGM/CGM administration.
Calabrese et al., 2022 [43], United States	Retrospective cohort; multicenter	Median, 64	4 (33.3)	IMIDs or IEI patients	412 IMIDs or IEI patients received TGM/CGM (IM) (150–150 mg dose) (*n* = 6), TGM/CGM (IM) (300–300 mg dose) (*n* = 6), and TGM/CGM (IM) dose was not reported (*n* = 400)	No comparator group	Median (IQR), 19 (13–84) after TGM/ CGM (IM) (150–150 mg dose) and Median (IQR), 38.5 (19–72) after TGM/ CGM (IM) (300–300 mg dose)	***RT-PCR positivity for SARS-CoV-2:*** 12/412 IMIDs and IEI patients developed COVID-19. ***Severity of COVID-19:*** eleven patients who developed COVID-19 following TGM/CGM combination were classified as mild and recovered at home. ***COVID-19-related hospitalization:*** 1/12 IMID or IEI patients required hospitalization. ***Mortality:*** 0 IMID or IEI patients died attributed to COVID-19.	One possible serious AE in a patient with COVID-19 with ITP.	NOS, 8	All cases who developed a breakthrough SARS-CoV-2 infection had been vaccinated against COVID-19.
Cochran et al., 2022 [42], United States	Retrospective cohort; single center	Not reported	Not reported	SOTRs	205 SOTR patients received TGM/CGM (IM) (150–150 mg dose (*n* = 14) or 300–300 mg dose (*n* = 191))	No comparator group	Not reported	***RT-PCR positivity for SARS-CoV-2:*** percentage of patients with positive SARS-CoV-2 PCR was higher in the TGM/CGM group who received the lower (150–150 mg) dose of TGM/CGM compared to those who received the higher dose of 300–300 mg (4/14 vs. 12/156). ***COVID-19-related hospitalization:*** 1/14 in the lower (150–150 mg) dose of TGM/CGM group compared to 2/156 in the higher (300–300 mg) dose of TGM/CGM required hospitalization. ***Mortality:*** 1/14 in the lower (150–150 mg) dose of TGM/CGM group compared to 1/156 in the higher (300–300 mg) dose of TGM/CGM died due to COVID-19.	5/205 developed cardiac events due to TGM/CGM combination: one event was an atrial fibrillation and other events were pericarditis, recurrent atrial flutter, mild/moderate cardiac allograft rejection, and complete heart block in a patient with history of LBBB.	NOS, 8	SOTRs received TGM/CGM during the period corresponding to the peak of the BA.2 and BA.5 (Omicron) wave in their region.
Conte et al., 2022 [23], United States	Retrospective cohort; single center	Median (IQR), 50 (27–72)	8 (44.4)	MS patients treated with OCR/OFA	18 MS patients received TGM/CGM (IM) (150–150 mg dose)	Same MS patients before receiving TGM/CGM (at baseline)	Mean, 14	***Antibody level:*** all patients had antibody level >250 U/mL. At baseline there were 12 patients lower than 0.8 U/mL and 6 higher than the threshold. After TGM/CGM, all 18 subjects were above the threshold (*p* < 0.001).	Not reported	NOS, 6	Study was completed prior to the FDA’s update to 300 mg each of TGM/CGM.
Davis et al., 2022 [41], United States	Retrospective cohort; single center	Median (IQR), 66 (18–91)	149 (59)	B-cell malignancies patients [CLL, DLBCL and MM]	251 B-cell malignancy patients received TGM/CGM (IM) (150–150 mg dose (*n* = 14) or 300–300 mg dose (*n* = 237))	No comparator group	Median (IQR), 91 (3–162)	***RT-PCR positivity for SARS-CoV-2:*** 27/251 developed COVID-19. ***COVID-19-related hospitalization:*** 4/27 had severe COVID-19 and required hospitalization. ***Mortality:*** 0 patients allocated to TGM/CGM combination died.	Two patients experienced diarrhea and rash. One patient with a history of epilepsy experienced a self-resolving seizure.	NOS, 8	Twenty-three infections occurred when Omicron variant BA.5 was dominant among the local population.
Fourati et al., 2022 [24], France	Retrospective case reports; single center	59 and 69	1 (33.3)	HSCTRs	3 HSCTR patients received TGM/CGM (IM) (150–150 mg dose)	No comparator group	9 and 11	***RT-PCR positivity for SARS-CoV-2:*** 2/3 developed COVID-19 (both cases were Omicron). ***Symptomatic COVID-19:*** 2/3 developed symptomatic COVID-19 in the TGM/CGM group. ***Severity of COVID-19:*** 2/3 who developed COVID-19 following TGM/CGM combination were classified as mild. ***COVID-19-related hospitalization:*** 2/3 required hospitalization.	Not reported	Modified NOS, moderate	Patients had a medical history of AML.
Goulenok et al., 2022 [25], France	Retrospective case-series; single center	Median (IQR), 52 (19–75)	4 (40)	IMIDs patients (AIDs and systemic vasculitis)	10 severely immunocompromised patients received TGM/CGM (IM) (150–150 mg dose)	7 severely immunocompromised patients received no mAbs	21	***RT-PCR positivity for SARS-CoV-2:*** positive PCR for SARS-CoV-2 was lower in the TGM/CGM group vs. no mAbs group (1 (Omicron, *n* = 1) vs. 7 (Omicron, *n* = 6 and Delta, *n* = 1)). ***Severity of COVID-19:*** severity of SARS-CoV-2 infection was lower in the TGM/CGM group compared to no mAbs group (0 vs. 4). ***COVID-19-related hospitalization:*** hospital admission was lower among TGM/CGM group vs. no mAbs group (0 vs. 5). ***Need for oxygen therapy:*** oxygen therapy requirement was lower among TGM/CGM group vs. no mAbs group (0 vs. 4). ***Mortality:*** 0 patients allocated to TGM/CGM and 1 patient allocated to no mAbs died.	No severe AEs were recorded for TGM/CGM in all patients.	Modified NOS, high	The sample size was small, and the study was conducted at a single center.
Kaminski et al., 2022 [12], France	Retrospective cohort; single center	Mean (SD), 60 (14.4)	204 (61.2)	KTRs	333 KTRs received TGM/CGM (IM) (150–150 mg dose)	97 KTRs received placebo	Not reported	***Symptomatic COVID-19:*** 41 developed symptomatic COVID-19 in the TGM/CGM group compared to 42 among those who received placebo (HR 0.011 [CI 95% 0.063–0.198]; *p* < 0.001). ***COVID-19-related hospitalization:*** hospital admission was lower among TGM/CGM group vs. placebo group (4 vs. 11, HR 0.046 [CI 95% 0.013–0.158]; *p* < 0.001). ***ICU admission:*** ICU admission in the TGM/CGM group was lower compared to patients in the placebo group (2 vs. 6, HR 0.045 [CI 95% 0.008–0.240]; *p* < 0.001). ***Mortality:*** 1 patient allocated to TGM/CGM and 2 patients allocated to placebo died [HR 0.076 (CI 95% 0.005–1.161); *p* = 0.066].	Not reported	NOS, 7	KTRs received TGM/CGM during the period corresponding to the peak of the Omicron wave in their region. Some patients (TGM/CGM group: *n* = 137 and placebo group: *n* = 43) were previously treated with CRM-IDM.
Karaba et al., 2022 [26], United States	Prospective cohort; single center	62.5 (57.7–68.5)	25 (41)	SOTRs (KTRs, HTRs, and LTRs)	61 SOTRs received: TGM/CGM (IM) (300–300 mg dose)	Same SOTRs patients before receiving TGM/CGM (at baseline)	Not reported	***Neutralization of COVID-19 Omicron variant:*** proportion of patients who received TGM/CGM exhibited higher neutralizing inhibition against the Omicron compared to the pre-TGM/CGM patients’ group (10/61 vs. 5/61, *p* = 0.06).	Reported reactions were mild or moderate and were more frequent after 300–300 mg dosing vs. 150–150 mg dosing.	NOS, 6	Patients received SARS-CoV-2 vaccines before receiving TGM/CGM as a 1st step protection.
Kertes et al., 2022 [27], Israel	Retrospective cohort; multicenter	TGM/CGM group: 40–59 (29.9%), 60–69 (28.6%), and 70–79 (30.5)	512 (62.1)	Immunocompromised patients	825 immunocompromised patients received TGM/CGM (IM) (150–150 mg dose)	4299 immunocompromised patients received no treatment	Not reported	***RT-PCR positivity for SARS-CoV-2:*** positive PCR for SARS-CoV-2 was lower in the TGM/CGM group vs. the non-TGM/CGM group (29 vs. 308, *p* < 0.001, (OR: 0.51, 95% CI: 0.30–0.84)). ***COVID-19-related hospitalization:*** percentage of patients admitted to hospital was lower among TGM/CGM group vs. the non-TGM/CGM group (1 vs. 27, *p* = 0.07; (OR: 0.08, 95% CI: 0.01–0.54)). ***Mortality:*** 0 patients allocated to TGM/CGM and 40 patients allocated to the non-TGM/CGM died, *p* = 0.005.	Not reported	NOS, 6	TGM/CGM group was more likely to have CVD, diabetes, HTN and CKD, and more likely to have been vaccinated against COVID-19 than those who never received TGM/CGM.
Kleiboeker et al., 2022 [28], United States	Retrospective case-series, single center	Median (IQR), 54 (52–54)	2 (66.7)	LTRs	77 LTRs received: TGM/CGM (IM) (150–150 mg dose)	No comparator group	1, 0, and 29	Not reported	In the TGM/CGM group, patients had myalgia (*n* = 3), arthralgia (*n* = 2), fatigue (*n* = 2), nausea and vomiting (*n* = 1), diarrhea (*n* = 1), intermittent fevers (*n* = 1), chills (*n* = 1), and malaise (*n* = 1).	Modified NOS, high	In the same cohort, 139 kidney and 101 liver transplant recipients received TGM/CGM without any reports of myalgia.
Lafont et al., 2022 [29], France	Retrospective case-series; single center	Median (IQR), 56 (44–63)	8 (53)	Immunocompromised patients	15 immunocompromised patients received TGM/CGM (IM) (150–150 mg dose)	10 immunocompromised patients received no treatment	Not reported	***Asymptomatic COVID-19:*** 2 developed asymptomatic COVID-19 in the TGM/CGM group compared to 2 among those who received no treatment. ***Symptomatic COVID-19:*** 4 and 4 developed fever and dyspnea in the TGM/CGM group compared to 5 and 3 in the no treatment group; however, cough prevalence was the same in both groups (6 vs. 9). ***Need for oxygen therapy:*** lower number required oxygen in the TGM/CGM group compared to the no treatment group (2 vs. 3). ***COVID-19-related hospitalization:*** percentage of patients admitted to hospital was lower among TGM/CGM group vs. no treatment group (3 vs. 3). ***ICU admission:*** lower number of patients in the TGM/CGM group required ICU admission compared to patients in the no treatment group (1 vs. 2). ***Mortality:*** 0 patients allocated to TGM/CGM and 2 patients allocated to no treatment died.	Not reported	Modified NOS, high	Most patients in the TGM/CGM group received at least 3 doses of SARS-CoV-2 vaccines or CRM/IDM.
Levin et al., 2022 [9], Multicounty	Randomized double-blind controlled trial; multicenter	Mean (SD), 53.6 (15)	1865 (53.9)	Immunocompromised patients	3460 patients received TGM/CGM (IM) (150–150 mg dose)	1737 patients received placebo (IM) 1.5 mL injections consecutively	Not reported	***RT-PCR positivity for SARS-CoV-2:*** positive PCR for SARS-CoV-2 was lower in the TGM/CGM group vs. placebo (8/3441 vs. 17/1731, RRR (95% CI) = 76.7 (46 to 90); *p* < 0.001). ***Severity of COVID-19:*** number of patients with severe or critical COVID-19 illness was lower in the TGM/CGM group vs. placebo (0/3441 vs. 5/1731). ***Mortality:*** 0 patients allocated to TGM/CGM and 2 patients allocated to placebo treatment died. ***Symptomatic COVID-19:*** 11 developed symptomatic COVID-19 in the TGM/CGM group compared to 31 among those who received placebo.	Most AEs were mild (761 vs. 369) or moderate (387 vs. 191) in intensity. Incidence of serious AEs was similar in the two groups (64 vs. 27). Most common AE was injection site reaction: *n* = 82 in the TGM/CGM group compared to *n* = 36 in the placebo group.	RoB 2, low risk of bias	SARS-CoV-2 variants: in the TGM/CGM group, 1 participant was infected with a SARS-CoV-2 B.1.351 (beta) variant, while 10 participants in the placebo group (5 participants with B.1.1.7_1 (an alpha subvariant) and 5 participants with B.1.617.2 (Delta)).
Najjar-Debbiny et al., 2022 [40], Israel	Retrospective cohort; multicenter	Mean (SD), 66.2 (13.7)	402 (57.2)	Immunocompromised patients	703 immunocompromised patients received TGM/CGM (IM) (150–150 mg dose)	2812 immunocompromised patients received no TGM/CGM	Not reported	***RT-PCR positivity for SARS-CoV-2:*** positive PCR for SARS-CoV-2 was lower in the TGM/CGM group vs. the non-TGM/CGM group (72 vs. 377, HR 0.75 [CI 95% 0.58–0.96]; *p* = 0.02). ***COVID-19-related hospitalization:*** percentage of patients who needed hospitalization due to COVID-19 was lower in the TGM/CGM group vs. the non-TGM/CGM group (7 vs. 67, HR 0.41 (CI 95% 0.19–0.89); *p* = 0.045).	Not reported	NOS, 8	Patients in the TGM/CGM group were matched by propensity score to patients in the non-TGM/CGM group (controls) in a 1:4 ratio.
Nguyen et al., 2022 [30], France	Retrospective cohort; multicenter	Mean (SD), 58.9 (20.7)	Not reported	Immunocompromised patients (SOTRs, hematologic malignancies, immunosuppressants, or IEI)	1112 immunocompromised patients received TGM/CGM (IM) (150–150 mg dose)	No comparator group	Median (IQR), 21 (13–36)	***RT-PCR positivity for SARS-CoV-2:*** 49 patients had confirmed infection (29/49 patients were Omicron). ***Severity of COVID-19:*** 43/49 cases were classified as mild disease, whereas 6/49 cases were classified as moderate to severe. ***COVID-19-related hospitalization****:* 10/49 patients required hospitalization. ***Need for oxygen therapy**:* 6/49 patients required oxygen therapy. ***Non-invasive ventilation:*** 2/49 patients required non-invasive ventilation. ***Mortality:*** 2/49 patients died.	Not reported	NOS, 7	Patients with confirmed SARS-CoV-2 infection < 5 days following TGM/CGM administration were excluded from the analyses.
Ocon et al., 2022 [39], United States	Retrospective cohort; single center	Mean (SD), 59 (15)	13 (30.2)	SARD patients (RA, AAV, other vasculitis, immune-mediated myositis, Sjögren disease, and SLE)	43 SARD patients received TGM/CGM (IM) (150–150 mg dose (*n* = 5) or 300–300 mg dose (*n* = 38))	No comparator group	Not reported	***RT-PCR positivity for SARS-CoV-2:*** 1/43 patients developed COVID-19. ***COVID-19-related hospitalization:*** 0 SARD patients required hospitalization.	Reported AEs included myalgia (*n* = 3), flu-like symptoms (*n* = 2), fever (*n* = 2), injection site pain (*n* = 1), and/or headache (*n* = 1).	NOS, 8	Thirty-five SARD patients had received SARS-CoV-2 vaccinations before receiving TGM/GM.
Ollila et al., 2022 [31], United States	Retrospective cohort; single center	Not reported	Not reported	Hematologic malignancy patients	25 hematologic malignancy patients received TGM/CGM (IM) (150–150 mg dose)	12 hematologic malignancies patients received no treatment	Not reported	***RT-PCR positivity for SARS-CoV-2:*** positive PCR for SARS-CoV-2 was lower in the TGM/CGM group vs. the non-TGM/CGM group (0 vs. 3, *p* = 0.007). ***Mortality:*** 0 patients allocated to TGM/CGM and 1 patient allocated to no treatment died.	Not reported	NOS, 6	Hematologic malignancy patients included any type of lymphoid, myeloid, or plasma cell malignancy.
Ordaya et al., 2022 [32], United States	Retrospective case-series; single center	Median (IQR), 57 (28.7–71.5)	2 (25)	Immunocompromised patients (hematological malignancies, AIDs, SOTRs, HSCTRs, and other immunocompromising conditions)	674 immunocompromised patients received TGM/CGM (IM) (150–150 mg dose)	No comparator group	Median (IQR), 2.5 (1–7)	***RT-PCR positivity for SARS-CoV-2:*** 8 patients developed COVID-19 (one case was Omicron). ***Severity and asymptomatic COVID-19:*** 6 cases were classified as mild disease, whereas 2 cases were classified as asymptomatic. ***Mortality:*** none of the 8 patients who developed COVID-19 following TGM/CGM combination died. ***Need for oxygen therapy:*** 1/8 patients required oxygen therapy. ***COVID-19-related hospitalization:*** 2/8 patients required hospitalization.	Not reported	Modified NOS, high	Seven patients had received COVID-19 vaccines.
Stuver et al., 2022 [33], United States	Prospective cohort; single center	Median (IQR), 62 (35–89)	Not reported	Hematologic malignancy patients	52 hematologic malignancy patients received TGM/CGM (IM) (150–150 mg dose (*n* = 30) or 300–300 mg dose (*n* = 22))	No comparator group	8 and 30	***Neutralization of COVID-19 Omicron variant:*** plasma from 10/22 patients who received TGM/CGM 300 mg dose achieved significantly higher neutralization of Omicron-RBD (*p* = 0.003) compared to single TGM/CGM 150 mg dose patients. ***RT-PCR positivity for SARS-CoV-2:*** 2 patients developed COVID-19 (both had received a single TGM/CGM 150 mg dose). ***Mortality:*** neither of the 2 patients who developed COVID-19 following TGM/CGM combination died. ***COVID-19-related hospitalization:*** neither of the 2 patients who developed COVID-19 following TGM/CGM combination required hospitalization. ***Severity of COVID-19:*** both patients who developed COVID-19 following TGM/CGM combination were classified as symptomatic.	Not reported	NOS, 7	Most common diagnosis was non-Hodgkin lymphoma. Nearly one-half were HSCTRs or received prior chimeric antigen receptor T cell therapy.
Totschnig et al., 2022 [38], Austria	Prospective cohort; single center	Mean (SD), 59.6 (15.1)	53 (59.5)	Immunocompromised patients (hematologic malignancy, AIDs, MS, IEIs, and SOTRs)	89 immunocompromised patients received TGM/CGM (IM) (150–150 mg dose)	No comparator group	Median, 40	***Antibody level:*** median antibody values 1 and 3 months after TGM/CGM were 3965 (*p* < 0.0001) and 1647 (*p* = 0.0007) binding antibody units/mL, respectively. ***RT-PCR positivity for SARS-CoV-2:*** 2/13 patients developed COVID-19. ***COVID-19-related hospitalization:*** 1/13 patients required hospitalization. ***Need for oxygen therapy:*** 0 patients required oxygen therapy.	Not reported	NOS, 8	Patients had been vaccinated against SARS-CoV-2 with a mean dose frequency of 3.7 times, mostly with mRNA vaccines.
Woopen et al., 2022 [34], Germany	Retrospective case-series; single center	Median (IQR), 58.5 (48.5–64.2)	4 (66.7)	MS patients	6 MS patients received TGM/CGM (IM) (150–150 mg dose)	No comparator group	No case developed COVID-19	***RT-PCR positivity for SARS-CoV-2:*** 0/6 patients developed COVID-19	No severe AEs were recorded for TGM/CGM in all patients.	Modified NOS, high	Six MS patients had received SARS-CoV-2 vaccines before start of TGM/CGM.
Young-Xu et al., 2022 [35], United States	Retrospective cohort; multicenter	Mean (SD), 67.4 (11)	1579 (91)	Immunocompromised patients (cancer, immunosuppressants, immunocompromised, and renal disease)	1733 immunocompromised patients received TGM/CGM (IM) (150–150 mg dose)	6354 immunocompromised patients received placebo	Not reported	***RT-PCR positivity for SARS-CoV-2:*** positive PCR for SARS-CoV-2 was lower in the TGM/CGM group vs. placebo (6 vs. 69, (HR 0.34; 95% CI, 0.13–0.87)). ***COVID-19-related hospitalization:*** percentage of patients admitted to hospital was lower among TGM/CGM group vs. placebo group (1 vs. 38, (HR 0.13; 95% CI, 0.02–0.99)). ***Mortality:*** 10 patients allocated to TGM/CGM and 99 patients allocated to placebo died (HR 0.36; 95%CI, 0.18–0.73).	Not reported	NOS, 8	A small proportion of patients who received TGM/CGM were not immunocompromised.
Zerbit et al., 2022 [37], France	Prospective cohort; single center	Median (IQR), 71 (63–78)	36/57 (63)	Hematologic malignancy patients	102 hematologic malignancy patients received TGM/CGM (IM) (150–150 mg dose)	236 hematologic malignancy patients received no TGM/CGM	Not reported	***RT-PCR positivity for SARS-CoV-2:*** positive PCR for SARS-CoV-2 was lower in the TGM/CGM group vs. the non-TGM/CGM group (5/102 vs. 52/236, *p* < 0.05).	Not reported	NOS, 8	Proportion of COVID-19 patients who were hospitalized was not different between those who received TGM/CGM or not.

Abbreviations: AEs, adverse events; AIDs, autoimmune diseases; AML, acute myeloid leukemia; ARDS, acute respiratory distress syndrome; AAV, anti-neutrophil cytoplasmic antibody-associated vasculitis; BMI, body mass index; CRM/IDM, casirivimab/imdevimab; CI, confidence interval; CKD, chronic kidney disease; CLL, chronic lymphocytic leukemia; COVID-19, coronavirus disease 2019; CVD, cardiovascular disease; DLBCL, diffuse large B-cell lymphoma; FDA, Food and Drug Administration; HIV, human immunodeficiency virus; HSCTRs, hematopoietic stem cell transplant recipients; HTN, hypertension; HTRs, heart transplant recipients; ICHs, immunocompromised hosts; ICU, intensive care unit; IEI, inborn errors of immunity; IM, intramuscular; IMIDs, immune-mediated inflammatory diseases; IQR, interquartile range; IMV, invasive mechanical ventilation; ITP, immune-mediated thrombocytopenia; IV, intravenous; KTRs, kidney transplant recipients; LBBB, left bundle branch block; LTRs, liver transplant recipients; mAbs, monoclonal antibodies; MM, multiple myeloma; mRNA, messenger ribonucleic acid; MS, multiple sclerosis; NOS, Newcastle–Ottawa Scale; OCR/OFA, ocrelizumab and ofatumumab; PrEP, pre-exposure prophylaxis; RA, rheumatoid arthritis; RBD, receptor-binding domain; RoB 2, Version 2 of the Cochrane risk-of-bias tool for randomized trials; RRI, relative risk increase; RRR, relative risk reduction; RT-PCR, real-time reverse transcription-polymerase chain reaction; SARD, systemic autoimmune rheumatic disease; SARS-CoV-2, severe acute respiratory syndrome coronavirus 2; SD, standard deviation; SLE, systemic lupus erythematosus; SOTRs, solid organ transplant recipients; TGM/CGM, tixagevimab/cilgavimab.

## Abbreviations

COVID-19: coronavirus disease 2019; CRM/IDM: casirivimab/imdevimab; KTRs: kidney transplant recipients; SOTRs: solid organ transplant recipients; PrEP: pre-exposure prophylaxis; SCT: hematopoietic stem cell transplant; RBD: receptor-binding domain; mAbs: monoclonal antibodies; HTRs: heart transplant recipients; LTRs: liver transplant recipients; TGM/CGM: tixagevimab/cilgavimab; SARS-CoV-2: severe acute respiratory syndrome coronavirus 2.

## References

[1] Prendecki M., Clarke C., Edwards H., McIntyre S., Mortimer P., Gleeson S., Martin P., Thomson T., Randell P., Shah A. (2021). Humoral and T-cell responses to SARS-CoV-2 vaccination in patients receiving immunosuppression. Ann. Rheum. Dis..

[2] Rusk D.S., Strachan C.C., Hunter B.R. (2021). Lack of immune response after mRNA vaccination to SARS-CoV-2 in a solid organ transplant patient. J. Med. Virol..

[3] Belsky J.A., Tullius B.P., Lamb M.G., Sayegh R., Stanek J.R., Auletta J.J. (2021). COVID-19 in immunocompromised patients: A systematic review of cancer, hematopoietic cell and solid organ transplant patients. J. Infect..

[4] Dumortier J., Duvoux C., Roux O., Altieri M., Barraud H., Besch C., Caillard S., Coilly A., Conti F., Dharancy S. (2021). COVID-19 in liver transplant recipients: The French SOT COVID registry. Clin. Res. Hepatol. Gastroenterol..

[5] Abkhoo A., Shaker E., Mehrabinejad M.-M., Azadbakht J., Sadighi N., Salahshour F. (2021). Factors predicting outcome in intensive care unit-admitted COVID-19 patients: Using clinical, laboratory, and radiologic characteristics. Crit. Care Res. Pract..

[6] Benotmane I., Velay A., Vargas G.G., Olagne J., Cognard N., Heibel F., Braun-Parvez L., Martzloff J., Perrin P., Pszczolinski R. (2022). A rapid decline in the anti-receptor binding domain of the SARS-CoV-2 spike protein IgG titer in kidney transplant recipients after tixagevimab-cilgavimab administration. Kidney Int..

[7] US Food and Drug Administration. Fact Sheet for Healthcare Providers: Emergency Use Authorization for Evusheld (Tixagevimab Co-Packaged with Cilgavimab). https://www.fda.gov/media/154701/download.

[8] Wise J. (2022). COVID-19: Evusheld is approved in UK for prophylaxis in immunocompromised people. BMJ.

[9] Levin M.J., Ustianowski A., De Wit S., Launay O., Avila M., Templeton A., Yuan Y., Seegobin S., Ellery A., Levinson D.J. (2022). Intramuscular AZD7442 (tixagevimab–cilgavimab) for prevention of COVID-19. N. Engl. J. Med..

[10] Al Jurdi A., Morena L., Cote M., Bethea E., Azzi J., Riella L.V. (2022). Tixagevimab/cilgavimab pre-exposure prophylaxis is associated with lower breakthrough infection risk in vaccinated solid organ transplant recipients during the omicron wave. Am. J. Transplant. Off. J. Am. Soc. Transplant. Am. Soc. Transpl. Surg..

[11] Bertrand D., Laurent C., Lemée V., Lebourg L., Hanoy M., Le Roy F., Nezam D., Pruteanu D., Grange S., de Nattes T. (2022). Efficacy of anti–SARS-CoV-2 monoclonal antibody prophylaxis and vaccination on the Omicron variant of COVID-19 in kidney transplant recipients. Kidney Int..

[12] Kaminski H., Gigan M., Vermorel A., Charrier M., Guirle L., Jambon F., Lacapère A., Ménard C., Moreau K., Neau-Cransac M. (2022). COVID-19 morbidity decreases with Tixagevimab/cilgavimab preexposure prophylaxis in kidney transplant recipients non/low vaccine responders. Kidney Int..

[13] Higgins J.P., Thomas J., Chandler J., Cumpston M., Li T., Page M.J., Welch V.A. (2019). Cochrane Handbook for Systematic Reviews of Interventions.

[14] Moher D., Liberati A., Tetzlaff J., Altman D.G., Group* P. (2009). Preferred reporting items for systematic reviews and meta-analyses: The PRISMA statement. Ann. Intern. Med..

[15] Peterson J., Welch V., Losos M., Tugwell P. (2011). The Newcastle-Ottawa scale (NOS) for assessing the quality of nonrandomised studies in meta-analyses. Ott. Ott. Hosp. Res. Inst..

[16] Bazerbachi F., Sawas T., Vargas E.J., Prokop L.J., Chari S.T., Gleeson F.C., Levy M.J., Martin J., Petersen B.T., Pearson R.K. (2018). Metal stents versus plastic stents for the management of pancreatic walled-off necrosis: A systematic review and meta-analysis. Gastrointest. Endosc..

[17] Sterne J.A., Savović J., Page M.J., Elbers R.G., Blencowe N.S., Boutron I., Cates C.J., Cheng H.-Y., Corbett M.S., Eldridge S.M. (2019). RoB 2: A revised tool for assessing risk of bias in randomised trials. BMJ.

[18] DerSimonian R., Kacker R. (2007). Random-effects model for meta-analysis of clinical trials: An update. Contemp. Clin. Trials.

[19] Egger M., Smith G.D., Schneider M., Minder C. (1997). Bias in meta-analysis detected by a simple, graphical test. BMJ.

[20] Benotmane I., Velay A., Gautier-Vargas G., Olagne J., Obrecht A., Cognard N., Heibel F., Braun-Parvez L., Keller N., Martzloff J. (2022). Breakthrough COVID-19 cases despite prophylaxis with 150 mg of tixagevimab and 150 mg of cilgavimab in kidney transplant recipients. Am. J. Transplant..

[21] Benotmane I., Velay A., Vargas G.G., Olagne J., Thaunat O., Fafi-Kremer S., Caillard S. (2022). Pre-exposure prophylaxis with 300 mg Evusheld™ elicits limited neutralizing activity against the Omicron variant. Kidney Int..

[22] Bruel T., Hadjadj J., Maes P., Planas D., Seve A., Staropoli I., Guivel-Benhassine F., Porrot F., Bolland W.-H., Nguyen Y. (2022). Serum neutralization of SARS-CoV-2 Omicron sublineages BA. 1 and BA. 2 in patients receiving monoclonal antibodies. Nat. Med..

[23] Conte W.L., Golzarri-Arroyo L. (2022). Tixagevimab and Cilgavimab (Evusheld) boosts antibody levels to SARS-CoV-2 in patients with multiple sclerosis on b-cell depleters. Mult. Scler. Relat. Disord..

[24] Fourati S., Robin C., Rodriguez C., Leclerc M., Beckerich F., Pawlotsky J.M., Redjoul R., Maury S. (2022). Breakthrough COVID-19 infections in vaccinated recipients of allogeneic stem cell transplantation. EJHaem.

[25] Goulenok T., Delaval L., Delory N., François C., Papo T., Descamps D., Ferré V.M., Sacré K. (2022). Pre-exposure anti-SARS-CoV-2 monoclonal antibodies in severely immunocompromised patients with immune-mediated inflammatory diseases. Lancet Rheumatol..

[26] Karaba A.H., Kim J., Chiang T.P.-Y., Alejo J.L., Abedon A.T., Mitchell J., Chang A., Eby Y., Johnston T.S., Aytenfisu T.Y. (2022). Omicron BA. 1 and BA. 2 Neutralizing Activity Following Pre-Exposure Prophylaxis with Tixagevimab plus Cilgavimab in Vaccinated Solid Organ Transplant Recipients. medRxiv.

[27] Kertes J., David S.S.B., Engel-Zohar N., Rosen K., Hemo B., Kantor A., Adler L., Stein N.S., Reuveni M.M., Shahar A. (2022). Association between AZD7442 (tixagevimab-cilgavimab) administration and SARS-CoV-2 infection, hospitalization and mortality. Clin. Infect. Dis..

[28] Kleiboeker H.L., Jorgenson M.R., Smith J.A. (2022). Myalgia in liver transplant recipients after receiving tixagevimab/cilgavimab for pre-exposure prophylaxis of COVID-19: A case series. Transpl. Infect. Dis. Off. J. Transplant. Soc..

[29] Lafont E., Pere H., Lebeaux D., Cheminet G., Thervet E., Guillemain R., Flahault A. (2022). Targeted SARS-CoV-2 treatment is associated with decreased mortality in immunocompromised patients with COVID-19. J. Antimicrob. Chemother..

[30] Nguyen Y., Flahault A., Chavarot N., Melenotte C., Cheminant M., Deschamps P., Carlier N., Lafont E., Thomas M., Flamarion E. (2022). Pre-exposure prophylaxis with tixagevimab and cilgavimab (Evusheld©) for COVID-19 among 1112 severely immunocompromised patients. Clin. Microbiol. Infect..

[31] Ollila T.A., Masel R.H., Reagan J.L., Lu S., Rogers R.D., Paiva K.J., Taher R., Burguera-Couce E., Zayac A.S., Yakirevich I. (2022). Seroconversion and outcomes after initial and booster COVID-19 vaccination in adults with hematologic malignancies. Cancer.

[32] Ordaya E.E., Beam E., Yao J.D., Razonable R.R., Vergidis P. (2022). Characterization of Early-Onset Severe Acute Respiratory Syndrome Coronavirus 2 Infection in Immunocompromised Patients Who Received Tixagevimab-Cilgavimab Prophylaxis. Open Forum Infectious Diseases.

[33] Stuver R., Shah G.L., Korde N.S., Roeker L.E., Mato A.R., Batlevi C.L., Chung D.J., Doddi S., Falchi L., Gyurkocza B. (2022). Activity of AZD7442 (tixagevimab-cilgavimab) against Omicron SARS-CoV-2 in patients with hematologic malignancies. Cancer Cell.

[34] Woopen C., Konofalska U., Akgün K., Ziemssen T. (2022). Case report: Variant-specific pre-exposure prophylaxis of SARS-CoV-2 infection in multiple sclerosis patients lacking vaccination responses. Front. Immunol..

[35] Young-Xu Y., Epstein L., Marconi V.C., Davey V., Zwain G., Smith J., Korves C., Cunningham F., Bonomo R., Ginde A.A. (2022). Tixagevimab/Cilgavimab for Prevention of COVID-19 during the Omicron Surge: Retrospective Analysis of National VA Electronic Data. medRxiv.

[36] Aqeel F., Geetha D. (2022). Tixagevimab and Cilgavimab (Evusheld©) in Rituximab-treated ANCA Vasculitis Patients. Kidney Int. Rep..

[37] Zerbit J., Detroit M., Meyer A., Decroocq J., Deau-Fischer B., Deschamps P., Birsen R., Mondesir J., Franchi P., Miekoutima E. (2022). Patients with Hematological Malignancies Treated with T-Cell or B-Cell Immunotherapy Remain at High Risk of Severe Forms of COVID-19 in the Omicron Era. Viruses.

[38] Totschnig D., Augustin M., Niculescu I., Laferl H., Jansen-Skoupy S., Lehmann C., Wenisch C., Zoufaly A. (2022). SARS-CoV-2 Pre-Exposure Prophylaxis with Sotrovimab and Tixagevimab/Cilgavimab in Immunocompromised Patients—A Single-Center Experience. Viruses.

[39] Ocon A.J., Mustafa S.S. (2022). Real-World Experience of Tixagevimab and Cilgavimab (Evusheld) in Rheumatologic Patients on Rituximab. JCR J. Clin. Rheumatol..

[40] Najjar-Debbiny R., Gronich N., Weber G., Stein N., Saliba W. (2022). Effectiveness of Evusheld in Immunocompromised Patients: Propensity Score-Matched Analysis. Clin. Infect. Dis..

[41] Davis J.A., Granger K., Roubal K., Smith D., Gaffney K.J., McGann M., Cendagorta A., Thurlapati A., Herbst A., Hendrickson L. (2022). Efficacy of tixagevimab-cilgavimab in preventing SARS-CoV-2 for patients with B-cell malignancies. Blood.

[42] Cochran W., Salto-Alejandre S., Barker L., Langlee J., Freed K., Carter D., Bannon J., Goddard D., Mostafa H., Werbel W. (2022). COVID-19 Outcomes in Solid Organ Transplant Recipients Who Received Tixagevimab-cilgavimab Prophylaxis and/or Bebtelovimab Treatment in a Nurse-driven Monoclonal Antibody Program during the Omicron Surge. Transplantation.

[43] Calabrese C., Kirchner E., Villa-Forte A., Hajj-Ali R.A., Moss B.P., Fernandez J.P., Calabrese L. (2022). Early experience with tixagevimab/cilgavimab pre-exposure prophylaxis in patients with immune-mediated inflammatory disease undergoing B cell depleting therapy and those with inborn errors of humoral immunity. RMD Open.

[44] Al-Obaidi M.M., Gungor A.B., Kurtin S.E., Mathias A.E., Tanriover B., Zangeneh T.T. (2022). The prevention of COVID-19 in high-risk patients using tixagevimab–cilgavimab (Evusheld): Real-world experience at a large academic center. Am. J. Med..

[45] Pelfrene E., Mura M., Sanches A.C., Cavaleri M. (2019). Monoclonal antibodies as anti-infective products: A promising future?. Clin. Microbiol. Infect..

[46] Taylor P.C., Adams A.C., Hufford M.M., De La Torre I., Winthrop K., Gottlieb R.L. (2021). Neutralizing monoclonal antibodies for treatment of COVID-19. Nat. Rev. Immunol..

[47] Zost S.J., Gilchuk P., Case J.B., Binshtein E., Chen R.E., Nkolola J.P., Schäfer A., Reidy J.X., Trivette A., Nargi R.S. (2020). Potently neutralizing and protective human antibodies against SARS-CoV-2. Nature.

[48] Planas D., Saunders N., Maes P., Guivel-Benhassine F., Planchais C., Buchrieser J., Bolland W.-H., Porrot F., Staropoli I., Lemoine F. (2022). Considerable escape of SARS-CoV-2 Omicron to antibody neutralization. Nature.

[49] Funk T., Pharris A., Spiteri G., Bundle N., Melidou A., Carr M., Gonzalez G., Garcia-Leon A., Crispie F., O’Connor L. (2021). Characteristics of SARS-CoV-2 variants of concern B. 1.1. 7, B. 1.351 or P. 1: Data from seven EU/EEA countries, weeks 38/2020 to 10/2021. Eurosurveillance.

[50] Hachmann N.P., Miller J., Collier A.-r.Y., Ventura J.D., Yu J., Rowe M., Bondzie E.A., Powers O., Surve N., Hall K. (2022). Neutralization escape by SARS-CoV-2 Omicron subvariants BA. 2.12. 1, BA. 4, and BA. 5. N. Engl. J. Med..

[51] Takashita E., Yamayoshi S., Simon V., van Bakel H., Sordillo E.M., Pekosz A., Fukushi S., Suzuki T., Maeda K., Halfmann P. (2022). Efficacy of antibodies and antiviral drugs against omicron BA. 2.12. 1, BA. 4, and BA. 5 subvariants. N. Engl. J. Med..

[52] Birabaharan M., Hill E., Begur M., Kaelber D.C., Martin T., Mehta S.R. (2022). Cardiovascular outcomes after tixagevimab and cilgavimab use for pre-exposure prophylaxis against COVID-19: A population-based propensity-matched cohort study. Clin. Infect. Dis..

[53] Kunutsor S.K., Laukkanen J.A. (2020). Incidence of venous and arterial thromboembolic complications in COVID-19: A systematic review and meta-analysis. Thromb. Res..

[54] Lodigiani C., Iapichino G., Carenzo L., Cecconi M., Ferrazzi P., Sebastian T., Kucher N., Studt J.-D., Sacco C., Bertuzzi A. (2020). Venous and arterial thromboembolic complications in COVID-19 patients admitted to an academic hospital in Milan, Italy. Thromb. Res..

[55] Piazza G., Campia U., Hurwitz S., Snyder J.E., Rizzo S.M., Pfeferman M.B., Morrison R.B., Leiva O., Fanikos J., Nauffal V. (2020). Registry of arterial and venous thromboembolic complications in patients with COVID-19. J. Am. Coll. Cardiol..

[56] Rabaan A.A., Bakhrebah M.A., Mutair A.A., Alhumaid S., Al-Jishi J.M., AlSihati J., Albayat H., Alsheheri A., Aljeldah M., Garout M. (2022). Systematic review on pathophysiological complications in severe COVID-19 among the non-vaccinated and vaccinated population. Vaccines.

[57] Hirsch C., Park Y.S., Piechotta V., Chai K.L., Estcourt L.J., Monsef I., Salomon S., Wood E.M., So-Osman C., McQuilten Z. (2022). SARS-CoV-2-neutralising monoclonal antibodies to prevent COVID-19. Cochrane Database Syst. Rev..

